# Adolescent perspectives on participating in a feasibility trial investigating shoe inserts for patellofemoral pain

**DOI:** 10.1186/s13047-022-00537-4

**Published:** 2022-05-16

**Authors:** Isobel C. O’Sullivan, Nathalia Cordeiro da Costa, Melinda M. Franettovich Smith, Bill Vicenzino, Kay M. Crossley, Steven J. Kamper, Marienke van Middelkoop, Hylton B. Menz, Kylie Tucker, Karina T. O’Leary, Natalie J. Collins

**Affiliations:** 1 School of Health and Rehabilitation Sciences: Physiotherapy, The University of Queensland, Brisbane, Australia; 2 La Trobe Sport and Exercise Medicine Research Centre, School of Allied Health, Human Services and Sport, La Trobe University, Melbourne, Australia; 3 Faculty of Medicine and Health, The University of Sydney, Sydney, Australia; 4 Nepean Blue Mountains Local Health District, Penrith, Australia; 5 Department of General Practice, Erasmus MC Medical University Center, Rotterdam, The Netherlands; 6 School of Allied Health, Human Services and Sport, La Trobe University, Melbourne, Australia; 7 School of Biomedical Sciences, The University of Queensland, Brisbane, Australia; 8 Surgical Treatment and Rehabilitation Services, Metro North Hospital and Health Service, Herston, Australia; 9 Faculty of Health and Behavioural Sciences, The University of Queensland, Brisbane, Australia

**Keywords:** Patellofemoral pain, Adolescents, Foot orthoses, Feasibility, Qualitative

## Abstract

**Background:**

Patellofemoral pain (PFP) affects one-quarter of adolescents, yet there are few evidence-informed recommendations to treat PFP in this population. HAPPi Kneecaps! is a randomised, controlled, participant- and assessor-blind, parallel-group feasibility trial of shoe inserts for adolescents with PFP. The aim of this qualitative study was to explore adolescents’ perspectives of participating in HAPPi Kneecaps!.

**Methods:**

All 36 adolescents with PFP from the HAPPi Kneecaps! study were invited to participate in semi-structured interviews. We used a descriptive qualitative methodology underpinned by a relativist framework to investigate adolescents’ perspectives on participating in the trial. Inductive thematic analysis was used to examine patterns regarding how each adolescent experienced the HAPPi Kneecaps! study within their social, cultural, and historical contexts.

**Results:**

14 out of 36 HAPPi Kneecaps! participants provided consent and participated in interviews (12 females; mean [SD] age 14.9 [2.4] years). Overall, most adolescents responded positively when discussing their experience, such as improvements in their knee pain and satisfaction with how the study was run. Major themes that were generated from the analysis and feedback were: (1) shoe inserts require little effort to use; (2) perceptions of the program were generally positive; (3) participation in the trial could be made easier; (4) warm weather matters; and (5) life happens.

**Conclusion:**

Adolescents with PFP who participated in the HAPPi Kneecaps! study found that shoe inserts were easy to wear. Most adolescents experienced an improvement in their symptoms and enhanced participation in sport and exercise. Adolescents with PFP prefer an option for warmer climates (e.g. flip flops or sandals), access to online logbooks, and clinicians who are easily accessible.

**Trial registration:**

Australian New Zealand Clinical Trials Registry (ANZCTR): ACTRN12619000957190. Date registered: 8/07/2019.

## Background

Patellofemoral pain (PFP), or pain around the kneecap, affects more than one-quarter of adolescents [1], and is one of the most common sources of musculoskeletal pain in this population [2]. Because pain during adolescence has detrimental effects on school attendance, physical and social activities, quality of life, appetite, sleep, and mental health [3–7], it is important that we identify effective strategies to manage PFP that are specific for this age group.

In contrast to the growing body of evidence for treating PFP in adults, very few studies have evaluated treatments in adolescents with PFP. Consensus-based guidelines recommend exercises targeting hip and knee muscles for adults with PFP, as well as exercise combined with patellar taping and mobilisation [8]. Adolescents tend to have poorer outcomes than adults when using this approach (38% success rate compared to 62–81% in adults) [9, 10]. This may be because adolescents have a higher prevalence of bilateral pain [10–12] and present with different impairments than adults [5, 9], or because they have lower adherence with exercise interventions [10].

Foot orthoses are simple inserts worn in everyday shoes and may be an effective intervention for adolescent PFP that addresses the unique characteristics of this group. A pilot study found that female adolescents reported greater improvement in PFP when soft foot orthoses were added to an exercise program, compared to flat shoe insoles [13]. However, there are no randomised controlled trials (RCTs) evaluating the efficacy of foot orthoses for adolescents with PFP. To address this evidence gap, we conducted the HAPPI Kneecaps! (sHoe inserts for Adolescents with Patellofemoral PaIn) study to determine the feasibility of conducting a full-scale RCT comparing contoured foot orthoses and flat shoe insoles in adolescents with PFP [14, 15]. We found that, for a full-scale RCT to be feasible, protocol modifications would be required to improve participant retention, completion of logbooks, and adherence with shoe insert wear [15].

To further inform the design of a full-scale RCT investigating shoe inserts for adolescents with PFP, we conducted a qualitative study to explore participants’ perspectives of the HAPPi Kneecaps! study [14]. The perspectives of adolescents with musculoskeletal conditions of participating in a trial, and of an intervention, have not been considered previously. Consumer input provides critical information to inform study design, such as core domains to evaluate [16], and can improve recruitment in clinical trials, especially when consumers with lived experience of the health condition are involved [17]. This is particularly important in the context of adolescents with PFP, who come with their own unique characteristics and perspectives that need to be considered. The aim of this study was to explore adolescents’ perspectives of participating in the HAPPi Kneecaps! study, including barriers and facilitators, to further inform feasibility of a full-scale RCT.

## Methods

We used a descriptive qualitative methodology with semi-structured interviews to investigate adolescents’ perspectives on participating in HAPPi Kneecaps! [14, 15]. Our study was underpinned by a relativist framework, which argues that reality is understandable through an individual’s knowledge and interpretation [18]. Congruent with this theoretical underpinning, we used inductive thematic analysis to analyse data gathered through the interviews and examined patterns within how individuals spoke about their experience of participating in the trial. This type of analysis is based on the individual’s understanding of their knee pain and participation in the trial within their social, cultural and historical contexts [18]. This paper discusses findings from the thematic analysis of participants’ responses, and follows the Consolidated Criteria for Reporting Qualitative Studies (COREQ) [19]. Ethics approval was obtained through The University of Queensland’s Human Research Ethics Committee (HREC No. 2018000159). The full protocol of the HAPPi Kneecaps! study is available in an earlier publication [14].

### Participants

We purposefully recruited adolescents who had PFP and had participated in the HAPPi Kneecaps! feasibility study [15]. Recruitment for HAPPi Kneecaps! was conducted in Brisbane, Australia, via social media platforms and advertisements within secondary schools, sporting clubs and at local community events, and from a database of adolescents with PFP who had volunteered in previous studies [14]. Volunteers were included in HAPPi Kneecaps! if they were aged 12–18 years and had anterior knee pain for at least 2 months (present during most weeks) of at least 3/10 severity (numerical rating scale), aggravated by activities that load the patellofemoral joint (e.g. squatting, stair ambulation). Volunteers were excluded if they had: (i) concomitant pain at sites other than the anterior knee; (ii) a history of knee, hip or spine surgery or other suspected knee joint pathology; (iii) planned lower limb surgery; or (iv) recent treatment for PFP. 36 HAPPi Kneecaps! participants were randomised to receive either contoured foot orthoses (*n* = 17) or flat shoe insoles (*n* = 19), fitted by a physiotherapist, and were followed over 3 months. All participants from both groups were invited to participate in this qualitative study. The final sample size for the qualitative study was determined when there appeared to be sufficient depth of information related to the study aims for a rigorous analysis and reporting [20].

### Data collection and procedure

Participants were interviewed once after the feasibility trial was completed, via an online video conferencing portal (Zoom Video Communications, California, USA). Each interview was recorded for transcription purposes. All participants provided verbal informed consent prior to the start of the interview. If adolescents were under 18 years of age, their parent/guardian also gave verbal consent. Interviews were conducted by one of two female physiotherapists (ICO, NC). ICO has 4 years’ experience working with patients experiencing musculoskeletal pain and was trained in qualitative research. NC has a PhD in physiotherapy, experience with qualitative research methods, and 9 years’ experience working with patients experiencing musculoskeletal pain. We acknowledge that the researchers’ clinical background, and levels of experience with qualitative research and research more broadly have shaped how the interviews were conducted, the data analysis and the findings produced.

**Fig. 1:**
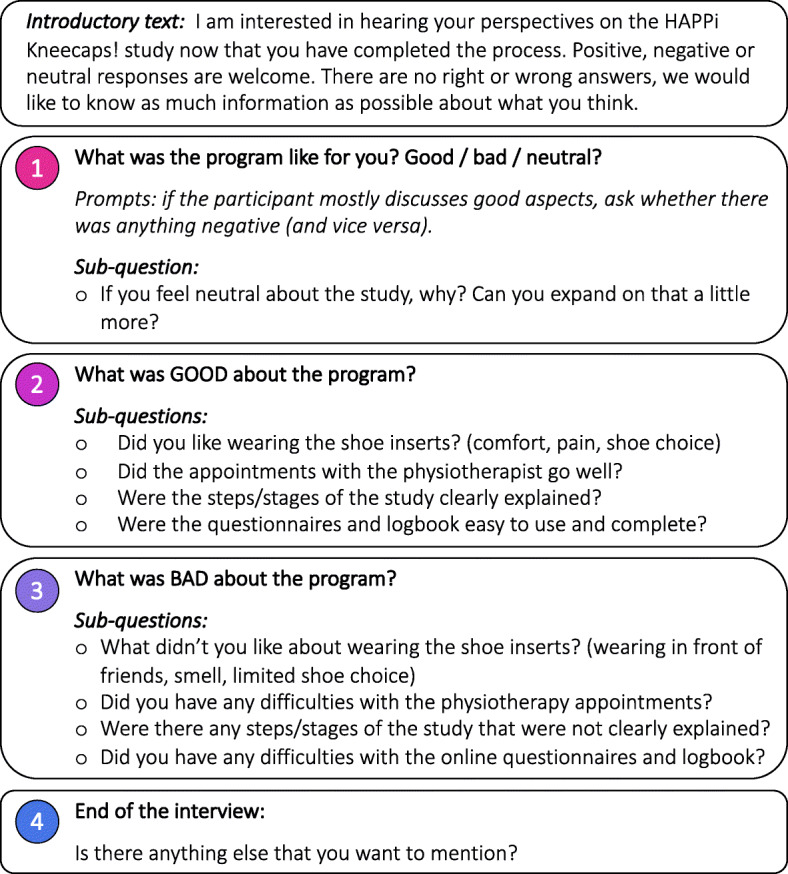
Interview guide

The interview guide was designed to encourage adolescents to discuss their perceptions and experiences of the study (positive, negative, or neutral) (Fig. 1). We asked adolescents to elaborate on their thoughts about the steps or stages of the study (physical screening, physiotherapy appointments, follow-up questionnaires). We also prompted them to discuss their experiences with the outcome measurement tools (online questionnaires, participant logbook) and physiotherapy appointments, likes and dislikes of the shoe inserts, barriers to adherence, impact of the shoe inserts and participation in the study on their life. Prior to data collection, the first author (IO) conducted a pilot interview with an adolescent with PFP who was not included in the feasibility study, with the view to identify potential issues related to the interview guide and the feasibility of conducting interview procedures over Zoom. After the pilot interview, members of the authorship team (IO, NJC, BV, MMFS) met to make minor adjustments to the interview guide (e.g. rewording questions to increase breadth). Data from the pilot interview were not included in the final analysis. Adolescents’ anonymity was maintained throughout transcription and data analysis by using pseudonyms. Each interview was transcribed verbatim by one investigator (ICO).

### Data analysis

Demographic and clinical data were summarised using descriptive statistics. Reflexive thematic analysis was conducted by two investigators (ICO, NC), following techniques described by Braun and Clarke [18, 21, 22]. First, the investigators read through the complete dataset independently and made notes on potential concepts and codes in relation to the research question, which were then discussed between the investigators. Second, each investigator reviewed the whole dataset and manually coded each interview using Microsoft Word. Third, codes were transferred into an Excel spreadsheet to analyse data and develop preliminary themes and codes. Fourth, coding was refined after the two investigators discussed discrepancies, and subsequently reviewed by additional members of the research team (NJC, BV, MMFS). To ensure trustworthiness and rigour within the data, we employed member checking and investigator triangulation. Member checking involved asking adolescents to report the main points discussed during the interview at its conclusion, and then restating the summarised information [23, 24]. Member checking was also conducted during the interview by restating comments made by adolescents. This approach was chosen to ensure that the information recorded was authentic and the views of adolescents were accurately represented [23, 24]. Triangulation involved the inclusion of authors with differing levels of clinical and research experience in the data analysis (ICO, NC, NJC, BV, MMFS). The final step of writing the results was essential to analysis and included the perspectives of all co-authors. Notably, the research team members were from a range of disciplines (physiotherapy, podiatry, movement control and pain) and brought their understandings of pain, experiences working with people in clinical practice, research perspectives and the socialisation to their discipline and profession into the analysis. Involving multiple researchers enables both confirmation of findings and consideration of different perspectives, adding breadth to the phenomenon of interest [25, 26].

## Results

All 36 participants from HAPPi Kneecaps! were approached to be involved in the qualitative study. 14 participants (39%; 12 [86%] females) who had completed the HAPPi Kneecaps! study agreed to participate in the interview. Adolescents in the qualitative study had a mean (SD) age of 14.9 (2.4) years, duration of symptoms of 24.8 (23.9) months, and baseline worst pain severity of 54.8 (24.9) mm (100 mm visual analogue scale; 0 = no pain, 100 = worst pain imaginable) (Table 1). Seven participants (50%) had bilateral PFP. Six participants (43%) were in the contoured foot orthoses group. Nine participants (64%) returned their logbooks showing an average wear time ranging between 1.2 and 8.1 h/ day over the 3-month period. Most participants received their inserts during Spring (64%). The number of appointments participants attended and the number of inserts they received varied. Interviews were conducted approximately 12 months after completion of the feasibility trial.

**Table 1:** Participant characteristics

Participant pseudonym	Age (years)	Assigned sex	Symptom duration (months)	Usual pain severity over the past week at baseline^**^**^	Worst pain severity over the past week at baseline^**^**^	PFP presentation	Group allocation	Season allocated*	Number of physiotherapy appointments attended	Number of pairs of inserts received	Average insert wear time (hours/day)^**#**^
Lucy	18	Female	72	40	40	Unilateral	Contoured	Spring	2	2	3.1
Lizzy	12	Female	12	10	55	Bilateral	Contoured	Spring	2	1	–
Katie	14	Female	8	50	70	Bilateral	Contoured	Winter	3	3	–
Zack	13	Male	3	25	41	Bilateral	Contoured	Summer	3	3	–
Fred	12	Male	3	65	85	Unilateral	Contoured	Spring	4	4	6.4
Mee	14	Female	2.5	60	75	Bilateral	Contoured	Spring	2	1	3.5
Rachel	17	Female	72	70	70	Unilateral	Flat	Spring	3	3	4.5
Marie	17	Female	48	2	3	Unilateral	Flat	Winter	3	2	4.3
Amy	18	Female	36	50	75	Unilateral	Flat	Spring	3	2	1.2
May	18	Female	24	13	15	Unilateral	Flat	Spring	2	2	4.0
Amelia	16	Female	24	60	80	Bilateral	Flat	Winter	3	3	–
Sally	13	Female	18	70	70	Bilateral	Flat	Winter	3	3	8.1
Jessica	15	Female	14	47	50	Bilateral	Flat	Spring	2	–	5.6
Lisa	12	Female	10	30	38	Unilateral	Flat	Spring	2	4	–

^^^ pain severity measured on a 100 mm visual analogue scale (0 = no pain, 100 mm = worst pain imaginable). # average daily wear time across 12-week study duration (as recorded in participant logbooks). ^*^ Average temperatures in Brisbane: Winter (10°-22° Celsius); Spring (14°-28° Celsius); Summer (21°-29° Celsius)

- data not available.

Table 2 provides an overview of the thematic analysis findings and codes within each theme. Overall, most adolescents responded positively when discussing their experience (e.g., reported reduced pain, were satisfied with how the study was conducted). The major themes that were generated from the analysis were: (1) shoe inserts require little effort to use; (2) perceptions of the program were generally positive; (3) participation in the trial could be made easier; (4) warm weather matters; and (5) life happens. We describe each theme below with quotes from participants, who are distinguished by pseudonyms to protect their confidentiality.

**Table 2:** Thematic analysis

Theme	Codes
***Shoe inserts require little effort to use***	- Not noticing the inserts was perceived as a good thing - The inserts were comfortable enough to not notice them - Inserts felt comfortable immediately - The intervention was straightforward as adolescents did not have to do much apart from keep the shoe inserts in their shoes - Having inserts fitted to school and sports shoes made it easy to wear them
***Perceptions of the program were generally positive***	- Best thing about the program: knee pain subsided - Belief that intervention helped PFP - Enhanced pain-free exercise participation - A reduction in symptoms is positive even when PFP is not completely recovered - The inserts were comfortable but did not help with the pain - Pain was still present throughout the whole study - Pain decreased in a few activities, but not in others
***Participation in the trial could be made easier***	- Questionnaires and logbook were straightforward, and clear - Logbook was simple - No difficulties with online questionnaires - Three shoes with inserts did not limit shoe choice - Potential barrier with travel to physiotherapy appointments - Hard to remember to fill in the logbook - Unsure about how much detail to include in logbook
***Warm weather matters***	- Summer affected the use of the inserts - Negative view of warm climate and having to wear closed-in shoes - Cold weather would have made adolescents more likely to wear the shoe inserts
***Life happens***	- Forgot logbook between houses - Grew out of shoes and inserts - Unsure if knee pain stopped because of inserts or “growing out of it” - Started exercising more which helped with pain - Confounding factors changed lifestyle (e.g. COVID-19)

### Theme 1: shoe inserts require little effort to use

*“Yeah, it was a bit different to walk around in at the beginning but after a couple of days I got used to it. And it wasn’t uncomfortable or anything, like getting used to any new shoes.” (May).*

Overall, adolescents thought the intervention was easy to implement. They often said the inserts were comfortable enough to not be noticeable and, like May, most adolescents adapted to them in less than a week. Some adolescents were still wearing the inserts at the time of the interview, 12 months after completing final follow-up questionnaires:

*“The orthotics that I got helped with my knee pain and even though it sometimes comes back after I do a lot of exercise, when I don’t wear them, or they don’t fit in my shoes, then I notice that it gets worse. I can also do more before they feel sore. Being a part of the study has definitely helped my knee pain.” (Mee).*

Mee’s quote highlights that the intervention was easy to adhere to long-term. Another key point discussed by Mee and others was that, whilst the pain did not subside completely, it reduced enough to notice a difference when not wearing the inserts, for example: *“It actually helped my knees a lot. I’ve stopped using them after a bit and it came back but it definitely helped the pain when I used them.”* (Katie).

Having inserts fitted to school and sports shoes made it easy to wear the intervention for long periods: “*The orthotics were in the school shoes, joggers and going out shoes. So, it wasn’t hard to fit them in ( …*) *it was easy to wear them*.” (Fred). Rachel also said it was an easy experience: “*Yeah, it was really easy, it was like just put the insoles in and then I forgot about them*.” (Rachel).

### Theme 2: perceptions of the program were generally positive

When adolescents were asked about what was positive and negative about their participation in the trial, a common positive was reduced knee pain:

Interviewer: *“Can you tell me a bit about what the program was like for you?”*

Lucy: *“I didn’t have any problems, like it [the intervention] was comfortable. And of course, probably the best thing was that my knee pain subsided.”*

Like Lucy, most adolescents related the intervention to reduction of their symptoms. Experiencing less knee pain seemed to be a motivator in choosing to continue to wear the inserts. For example, Marie said:

*“I also stopped wearing them for a few weeks and my knees got sorer, so I put them back in and it made a big difference.”*

Overall, adolescents did not have major concerns about wearing the inserts, and often commented that the inserts enhanced their participation in physical activity and sport. Some adolescents mentioned that their symptoms had improved enough to participate in a sport previously precluded due to pain:

*“The pain has lessened quite a bit and like when I am doing sport, it’s not there.” (Jessica).*

Adolescents mentioned that an improvement in their symptoms was positive even if they were not completely recovered:

*“The pain’s not as bad as it was before [the study], like I would say it’s helped a lot, I’ve noticed a big improvement.” (Katie).*

Lizzy discussed how wearing the inserts allowed her to participate in a wider variety of sports:

*“I am able to participate in a lot more activities because I don’t have to worry about pain, and I was able to join other sports teams that required you to do more on your knees and feet ( …) which is good.” (Lizzy).*

Lizzy also said that she no longer had to modify her exercises when playing cricket:

*“In cricket you require both your lower and upper part of your body, and we had programs to work on each area and I would only do the batting one cause it was easier cause like I didn’t use my legs, but after I got them [the inserts] I was able to do the bowling ones cause you have to have like very strong knees so you’re able to do like bowl better so you’re able to get your run up better, and I would notice the pain when I jumped but after I got the inserts I was able to do it without pain.” (Lizzy).*

The above feedback indicates that symptom severity, for some adolescents, was reduced to the extent that they were able to participate in more activities with reduced or absent pain. Interestingly, one of the adolescents gave a pair of inserts to a friend who was experiencing similar symptoms, indicating that they felt the intervention was worth recommending to others:

*“They were comfortable enough and didn’t really notice they were there so I can’t really fault them or anything. The physio gave me an extra pair of the inserts and I ended up giving them to my friend with the same type of knee pain ( …*). *Well, I thought if it worked for me then she could use them too.”* (Lucy).

One adolescent found the inserts also made it easier to participate in everyday activities.

*“I’m able to walk further and I’m able to leave later because I don’t have to stop as much from the pain in my knee. I’m getting support that my feet need because I have to walk long distances to school, and I have to walk on several campuses to school so it was really nice to start getting to class quicker and on time.”* (Lizzy).

Conversely, some adolescents indicated that the intervention did not help. One adolescent thought their pain improved spontaneously or for other reasons, such as increased exercise. For instance: *“To be quite frank I think it was the workouts that helped the pain because I didn’t wear the shoes much.”* (Amy). Another mentioned that the intervention worsened their pain:

Interviewer: *“How did you find the program overall?”*

Zack: *"Bad because the knee pain has gotten worse, feel it all the time and the knee locks and grinds when bending and unbending it."*Of note is a comment by Zack’s mother, who was present during the interview and said that Zack did not wear the inserts much as the trial was during COVID-19 restrictions when he was home-schooling. Although there was some discrepancy between what Zack and his mother reported, it is important to acknowledge that he may have experienced the described symptoms whilst wearing the inserts as opposed to when not wearing them.

### Theme 3: participation in the trial could be made easier

Overall, the adolescents indicated that participation in the trial was straightforward, and questionnaires were easy to follow: *“It was really straightforward. The questionnaires were clear. And the physio I had was really nice and yeah it was really easy.”* (Rachel). Likewise, Sally thought that none of the processes involved in the trial were difficult: *“The logbook was like really easy to fill out and it was easy to keep track of and all the appointments and it made sense and it was easy to understand.”.*

One adolescent found the Wong–Baker Faces Pain Rating Scale in the logbook practical, and appreciated being able to track her activity and pain levels:

*“Yeah, it was easy cause they had diagrams for the pain scale and if I was unsure of the pain level, then I would look at all the faces that were on there. It was also easy for me to see if I did more exercise than usual; did it flare up or go away and depending on what shoes I wore.” (Mee).*

Although most adolescents discussed their participation in the trial as an “easy” experience, some adolescents provided indirect or direct suggestions as to what would improve the process for the future, such as the timing of appointments, length of questionnaires and method of distribution for outcome measures. One of the most frequent barriers was travel distance to physiotherapy appointments: *“It was a bit of a drive to get to [Suburb 1] from [Suburb 2], so everything was a bit of a hike.”* (Approximately 25 min travel) (Amelia’s mum).

Adolescents also reported that, if they had been provided more options for responding to follow up measures (e.g. via different platforms) and had been sent daily reminders to fill out the logbook, adherence would have been easier: *“A reminder would have helped me. Each night I had an alarm in my phone for after dinner to remember to fill it in [the logbook] and I had it in a set spot, so I remembered where it was.”* (Sally). Several adolescents indicated a preference for online logbooks: *“If they were online, it would have helped me keep up with it more.” (May).*

In addition, being given less than three pairs of shoe inserts was a barrier for some adolescents: *“I had Converse, my Docs [Dr. Martens leather shoe/boot] and my sports shoes and taking them out and putting them in other shoes was annoying. Having 3 pairs instead would be better.”* (Lucy). Older adolescents (17–18 years old) tended to wear a wider range of shoes throughout the week: *“I normally wear lots of different shoes so having more pairs [of inserts] would have made things even easier.”* (Rachel, age 17).

### Theme 4: warm weather matters

The trial was held in Brisbane (humid subtropical climate, Koppen climate classification: Cfa https://www.britannica.com/science/Koppen-climate-classification/World-distribution-of-major-climatic-types#ref284656) over a period of nine months (spring, summer, autumn), and inserts were to be worn in enclosed shoes that adolescents wore throughout the week. Most adolescents indicated that climate had an impact on their adherence with wearing the inserts, and that if they had been given a choice of flip flops or open shoes, they would have preferred them:

*“When I didn’t wear them [the inserts], it was uncomfortable but sometimes I felt that it was too hot to wear socks and shoes. It wasn’t that much trouble, but I just had to decide whether I wanted to have sweaty feet or have pain in my knees.” (Mee).*

For adolescents involved in the trial over winter, adherence was less of a concern. Lucy was given her intervention over summer and reported that she was wearing the inserts more after the trial was over when the climate was cooler:

*“Yeah, so I know that it was over summer, and I tend to use my flip flops. The shoes I put inserts in are sports shoes or Docs [Dr. Martens leather shoe/boot] and when it’s hot you don’t want to wear those kinds of shoes. Now, I’m probably wearing my inserts pretty much every day whereas back when the study was on, I was probably wearing them about 70% of the time. If the study had been done in winter, where the question was – how long did you wear your inserts today? – mine would have been yes probably like 95% of the time.” (Lucy).*

### Theme 5: life happens

Adolescents found that certain life factors affected their participation in the trial. One adolescent reported living between two houses due to separated parents and perceived this as a barrier to remembering to fill in the logbook. Lucy expressed a preference for an online logbook due to this issue: *“I remember having to find a pen or because I move between two houses, sometimes I’d forget it [the logbook].”* (Lucy).

Two adolescents grew out of their shoes during the study, affecting insert usage: *“I started off with one pair of shoes and actually grew out of them during the study so I started wearing different shoes which may not have fitted the inserts properly.”* (Zack). Likewise, Sally reported *“They [the inserts] felt fine but I just I wasn’t sure if they fit right anymore because I grew out of my shoes.”* Consequently, these adolescents stopped wearing the inserts.

Another factor considered important by adolescents was the pandemic. COVID-19 resulted in lifestyle changes and reduced time wearing shoes because participants spent more time at home. Amy reported doing more exercise during COVID-19 restrictions, which she believed helped her knee pain: *“To be quite frank, I’m not actually sure that the pain got better because of the orthotics like I’m still not quite sure, because I did change like quite a lot of lifestyle habits as the study progressed.”* (Amy). Zack also reported that home-schooling meant that he was not wearing shoes as often as usual: *“I was given 3 pairs of inserts, so all my shoes had the inserts in them, but I hardly wore my shoes during the period because of COVID.”*

## Discussion

Overall, adolescents with PFP who participated in the HAPPi Kneecaps! trial found that the shoe inserts were easy to wear in daily life. The major factors that influenced their experience in the trial were shoe insert comfort and ease of use, ease of logbook and online questionnaire completion, access to study physiotherapists, improvement in symptoms, and ability to participate in activities of daily life and sport. Importantly, adolescents highlighted key areas to improve for future studies, including the method of logbook completion, travel distances to physiotherapy clinics, shoe insert options for warmer climates, and accommodating growth spurts during the study (e.g., refitting inserts to larger shoes).

Adolescents reported that they found the inserts generally easy to use, not noticeable when worn, and immediately comfortable. This aligns with the prescription algorithm used in this study, where we fit the inserts primarily based on comfort [14]. Some adolescents, like Mee, were still using the inserts 12 months after the study had concluded. Together, this suggests that adolescents with PFP may be willing to continue wearing shoe inserts long-term for ongoing management of their knee pain, which is important given that adolescent PFP tends to persist into adulthood [27]. Improvement in symptoms following the use of shoe inserts may also increase adolescents’ adherence with targeted exercise therapy. Although full-scale clinical trials are required to establish efficacy, shoe inserts may be useful as an initial treatment to facilitate pain-free exercise in this population.

Most adolescents reported a positive perception of the trial, which arose from an improvement in symptoms that allowed greater participation in activities and sport. This is an important finding. Over two years, 71% of adolescents with PFP reduced or ceased physical activity participation due to PFP symptoms [28]. Physical activity in adolescence is a strong predictor of future physical activity [29] and a key determinant of overweight and obesity [30]. Therefore, identifying interventions that can help to maintain or improve physical activity participation in adolescents with PFP is crucial, and should be a focus of future studies. Interestingly, adolescents in our study had a positive perception of the trial even if symptoms were only reduced and not eliminated. Ease of use of shoe inserts is important to give the intervention the opportunity to improve symptoms, which may then facilitate longer-term adherence and other flow-on benefits, such as increased participation in sport and physical activity. This may be enhanced by addressing other barriers to wearing the inserts that adolescents identified (e.g., providing appropriate options for warm weather). While these implications are important, it is also notable that symptom improvements varied between adolescents, and a few participants experienced no improvement or worsening of their symptoms. Therefore, clinicians should consider that shoe inserts may not be appropriate for some adolescents, potentially due to lifestyle issues and other contextual factors.

Our third theme provides insight into factors affecting data collection and adherence to the interventions, which should be considered in future studies involving adolescents. For instance, opinions of the logbook varied, with some adolescents highlighting them as simple and straightforward, while others found it hard to know how much detail to include and to remember to complete it. Future trials should consider giving adolescent participants the option of an online or app-based logbook (rather than paper-based), sending daily reminders for logbook completion (e.g., text messages or push notifications), and providing more detailed guidance on how to complete the logbooks. Mee mentioned how tracking her activity and exercise via logbook recording made her more aware of specific exercises and activities that triggered PFP flare-ups. While being aware of trajectories and potential triggers may be helpful for some adolescents, it could also enhance fear or reluctance around certain activities and inhibit participation in others. A measure of pain-related fear (e.g., Modified Fear-Avoidance Beliefs Questionnaire-Physical Activity, Tampa Scale for Kinesiophobia-11) or pain catastrophizing (e.g., Pain Catastrophizing Scale-Child) could be considered in future studies to assess maladaptive beliefs in this population [31]. Drawing attention to certain behaviours that adolescents believe exacerbate their pain (e.g., sport or physical activity), could lead to fear-avoidance behaviour, hypervigilance, and safety-seeking behaviours that, in turn, affect outcomes [32].

### Lessons learnt

Adolescents were encouraged to attend three appointments with their study practitioner for fitting of up to four pairs of shoe inserts [14]. However, 43% of adolescents we interviewed only attended two appointments. The parent of one adolescent said that the distance to the physiotherapy clinic was too far to travel. In planning a full-scale RCT in this population, having study practitioners in a wider range of locations with opening hours that include evenings and weekends would improve accessibility for adolescents. This is particularly important for this age group, where time is required not only from the adolescent, but also their parent or guardian to assist with transport and attend practitioner appointments (where appropriate). Telehealth could also be considered for follow-up appointments where fitting of new shoe inserts or adjustments to existing inserts are not required. The number of appointments that adolescents attended may have influenced the number of inserts they received. Those who received fewer pairs of inserts reported that this was inconvenient and negatively impacted their adherence with wearing the inserts. Another factor affecting adherence with wearing the inserts was warm weather, with adolescents preferring to wear open shoes such as sandals and flip flops that did not accommodate the inserts. Shoe inserts may be ideal for times of the year when adolescents more frequently wear enclosed shoes (e.g., school shoes during semester, sports shoes during sports season, enclosed shoes during cooler months). Alternative footwear should be considered to maximise adherence during school holidays and warmer weather, particularly for humid sub-tropical climates. For example, future studies could consider providing flip flops with a contoured foot bed to adolescents allocated to receive contoured foot orthoses, while flip flops with a flat foot bed could be used for those assigned flat shoe insoles.

A common suggestion made during the interviews was for an online logbook in place of the paper logbook used in this study. Some adolescents reported that the paper logbook was cumbersome and difficult to remember to complete. One adolescent, who lived between two houses with separated parents, reported that they often forgot to take the logbook with them. An online and/or app-based logbook, with electronic reminders to enter daily data, could address some of these barriers, minimising missing data in future trials while also allowing participants to promptly report adverse events or problems with the intervention. The potential benefit of an online logbook in this population was demonstrated by the generally positive feedback and adherence with filling out the online questionnaires at 6 weeks and 3 months.

### Uncontrollable factors

Several uncontrollable factors contributed to adherence and the adolescents’ experience during the trial. One of the most common codes arising was the impact of the COVID-19 pandemic. COVID-19 restrictions meant that adolescents were undergoing home schooling, and normal sports and physical activities were curtailed. This had two effects. First, adolescents were not wearing shoes as frequently as they would during normal conditions. Second, some adolescents found it difficult to rate their pain when not engaging in their usual activities. Only one adolescent (Amy) described exercising more during the lockdown and believed her this was the reason her knee pain reduced rather than due to wearing the inserts. Another factor to consider is the belief of one adolescent that their knee pain subsided because they “grew out of it”, rather than because of the intervention. Further qualitative studies could be conducted to understand decision-making around pain and pain beliefs in this population to better equip clinicians to communicate with this group. Two adolescents had growth spurts during the trial and had to buy new shoes, meaning that the inserts no longer fit properly within the shoes. In a full-scale RCT, additional appointments should be organised during the trial for re-sizing, refitting and replacement of inserts as needed.

### Strengths and limitations

Building on findings of our feasibility study [15], this nested qualitative study provides detailed insight from participants to inform the design of a future full-scale RCT of shoe inserts in adolescents with PFP, particularly regarding aspects that may impact on feasibility. A limitation warranting attention is that, based on response from HAPPi Kneecaps! participants, our sample only includes those who completed 3-month follow-up. Although we made every effort to contact participants who dropped out of the feasibility trial to participate in the qualitative study, none responded. Therefore, perspectives on the study (including reasons for dropping out) were not obtained from adolescents who did not complete the study. It is important to note that participants’ perspectives and factors affecting retention over a period of longer than 3 months remains unknown, and should be considered when designing future full-scale RCTs with longer follow-up (e.g. 12 months). In addition, the population included in the HAPPi Kneecaps! trial, and subsequently this qualitative study, may not represent the general population of adolescents with PFP, in terms of socioeconomic status, cultural background, and race. It is likely that including a broader population would provide important additional insights to inform the design of a full-scale RCT for this population. This study was conducted in Southeast Queensland, Australia, where the warm, humid climate influenced footwear choices among adolescents and adherence to wearing the shoe inserts, which affects the degree to which the outcomes of this study are generalisable to all adolescents with PFP. Finally, because of COVID-19 delays in research, the interviews for this study were conducted approximately 12 months after the feasibility trial concluded. This gives rise to possible recall issues and may affect the accuracy of feedback about barriers and facilitators experienced during the trial.

## Conclusion

Overall, adolescents with PFP who participated in the HAPPi Kneecaps! feasibility trial found the shoe inserts easy to wear. Adolescents generally reported an improvement in their symptoms, and that wearing the inserts enhanced their participation in sport and exercise. Key considerations in the design of a future full-scale RCT of shoe inserts for adolescents with PFP include an option for warmer climates (e.g. flip flops or sandals), access to online data collection tools, and conveniently located clinics for fitting of their shoe inserts.

## Data Availability

De-identified individual participant data was collected during the trial. Access to this data will be determined on a case-by-case basis at the discretion of the Principal Investigator, after publication of the study, with a requirement to sign a data access agreement.

## Abbreviations

COREQ: Consolidated Criteria for Reporting Qualitative Studies
COVID-19: Coronavirus disease 2019
HAPPi Kneecaps!: sHoe inserts for Adolescents with Patellofemoral PaIn
PFP: Patellofemoral pain
RCT: Randomised controlled trial
SD: Standard deviation
